# Evaluation of Population-Level Changes Associated With the 2021 US Preventive Services Task Force Lung Cancer Screening Recommendations in Community-Based Health Care Systems

**DOI:** 10.1001/jamanetworkopen.2021.28176

**Published:** 2021-10-12

**Authors:** Debra P. Ritzwoller, Rafael Meza, Nikki M. Carroll, Erica Blum-Barnett, Andrea N. Burnett-Hartman, Robert T. Greenlee, Stacey A. Honda, Christine Neslund-Dudas, Katharine A. Rendle, Anil Vachani

**Affiliations:** 1 Institute for Health Research, Kaiser Permanente Colorado, Aurora; 2 Department of Epidemiology, University of Michigan, Ann Arbor; 3 Marshfield Clinic Research Institute, Marshfield, Wisconsin; 4 Center for Integrated Healthcare Research, Kaiser Permanente Hawaii, Oahu; 5 Henry Ford Health System and Henry Ford Cancer Institute, Detroit, Michigan; 6 Perelman School of Medicine, University of Pennsylvania, Philadelphia

## Abstract

**Importance:**

The US Preventive Services Task Force (USPSTF) released updated lung cancer screening recommendations in 2021, lowering the screening age from 55 to 50 years and smoking history from 30 to 20 pack-years. These changes are expected to expand screening access to women and racial and ethnic minority groups.

**Objective:**

To estimate the population-level changes associated with the 2021 USPSTF expansion of lung cancer screening eligibility by sex, race and ethnicity, sociodemographic factors, and comorbidities in 5 community-based health care systems.

**Design, Setting, and Participants:**

This cohort study analyzed data of patients who received care from any of 5 community-based health care systems (which are members of the Population-based Research to Optimize the Screening Process Lung Consortium, a collaboration that conducts research to better understand how to improve the cancer screening processes in community health care settings) from January 1, 2010, through September 30, 2019. Individuals who had complete smoking history and were engaged with the health care system for 12 or more continuous months were included. Those who had never smoked or who had unknown smoking history were excluded.

**Exposures:**

Electronic health record–derived age, sex, race and ethnicity, socioeconomic status (SES), comorbidities, and smoking history.

**Main Outcomes and Measures:**

Differences in the proportion of the newly eligible population by age, sex, race and ethnicity, Charlson Comorbidity Index, chronic obstructive pulmonary disease diagnosis, and SES as well as lung cancer diagnoses under the 2013 recommendations vs the expected cases under the 2021 recommendations were evaluated using χ^2^ tests.

**Results:**

As of September 2019, there were 341 163 individuals aged 50 to 80 years who currently or previously smoked. Among these, 34 528 had electronic health record data that captured pack-year and quit-date information and were eligible for lung cancer screening according to the 2013 USPSTF recommendations. The 2021 USPSTF recommendations expanded screening eligibility to 18 533 individuals, representing a 53.7% increase. Compared with the 2013 cohort, the newly eligible 2021 population included 5833 individuals (31.5%) aged 50 to 54 years, a larger proportion of women (52.0% [n = 9631]), and more racial or ethnic minority groups. The relative increases in the proportion of newly eligible individuals were 60.6% for Asian, Native Hawaiian, or Pacific Islander; 67.4% for Hispanic; 69.7% for non-Hispanic Black; and 49.0% for non-Hispanic White groups. The relative increase for women was 13.8% higher than for men (61.2% vs 47.4%), and those with a lower comorbidity burden and lower SES had higher relative increases (eg, 68.7% for a Charlson Comorbidity Index score of 0; 61.1% for lowest SES). The 2021 recommendations were associated with an estimated 30% increase in incident lung cancer diagnoses compared with the 2013 recommendations.

**Conclusions and Relevance:**

This cohort study suggests that, in diverse health care systems, adopting the 2021 USPSTF recommendations will increase the number of women, racial and ethnic minority groups, and individuals with lower SES who are eligible for lung cancer screening, thus helping to minimize the barriers to screening access for individuals with high risk for lung cancer.

## Key Points

**Question:**

Is the updated US Preventive Services Task Force (USPSTF) recommendations for lung cancer screening associated with a clinically meaningful change in the distribution of the characteristics of individuals who are eligible for screening?

**Findings:**

In this cohort study using data derived from 5 health care systems, the updated 2021 USPSTF recommendations were associated with an increased overall proportion of women, racial and ethnic minority groups, and individuals with lower socioeconomic status who are eligible for lung cancer screening.

**Meaning:**

The 2021 USPSTF recommendations for lung cancer screening eligibility are expected to enhance opportunities for community-based programs to reduce barriers to lung cancer screening access for individuals who are at highest risk for lung cancer.

## Teaser

This cohort study examines the latest expansion of the lung cancer screening eligibility criteria for adults in the US.

## Introduction

On March 9, 2021, the US Preventive Services Task Force (USPSTF) released guidelines that updated its 2013 recommendations for annual lung cancer screening with low-dose computed tomography and lowered the screening starting age from 55 to 50 years and minimum smoking history from 30 to 20 pack-years. The 2013 USPSTF recommendations were informed by criteria used in the National Lung Screening Trial (NLST), which reported that screening with low-dose computed tomography reduced lung cancer mortality by 20% in trial participants who were at an increased risk for lung cancer.^1^ In response to the NLST findings and 2013 USPSTF recommendations, community health systems across the United States began implementing lung cancer screening programs. Estimates of the uptake of lung cancer screening, however, remain low, and early experience suggests that the outcomes associated with lung cancer screening in community settings may differ from those experienced by NLST participants in part because of differences in the characteristics of the individuals who receive care in community health care systems.^2,3,4,5,6^

Previous research also suggested that when the 2013 USPSTF recommendations were applied in community health care settings, they may have exacerbated existing disparities in lung cancer diagnosis and outcomes, particularly among women, racial and ethnic minority groups, and those in the lowest socioeconomic status (SES) categories.^7,8,9,10^ Analyses conducted by the Cancer Intervention and Surveillance Modeling Network (CISNET) Lung Cancer Working Group estimated that the 2021 USPSTF recommendations could increase the screening-eligible population by 87% as well as the relative proportion of women by 96%, non-Hispanic Black individuals by 106%, Hispanic individuals by 112%, and Asian individuals by 61%.^11^ Moreover, CISNET estimated that the number of screening-detected lung cancers could increase by 21%.^11^

The CISNET models and the systematic evidence review that informed the 2021 USPSTF recommendations were both based largely on clinical trial findings.^11,12^ However, it is unknown how these changes in recommendations will affect community-based screening programs that serve diverse populations in heterogeneous settings. The estimated population impact of the 2021 USPSTF recommendations that was calculated by CISNET was also based on survey data, which were limited by potential sampling bias and misclassification of diagnostic vs screening scans.^13,14^ In this cohort study, we estimated the population-level changes associated with the 2021 USPSTF expansion of lung cancer screening eligibility by age, sex, race and ethnicity, sociodemographic factors, and comorbidities within 5 community-based health care systems. In addition, we compared the proportion of individuals with an incident lung cancer diagnosis under the 2013 USPSTF recommendations with the estimated number of incident cases under the 2021 USPSTF criteria.

## Methods

### Study Population and Setting

This cohort study was conducted within the Population-based Research to Optimize the Screening Process (PROSPR) Lung Consortium, a collaboration of 5 diverse health care systems in the US (Henry Ford Health System, Detroit, Michigan; Kaiser Permanente Colorado, Aurora, Colorado; Kaiser Permanente Hawaii, Oahu, Hawaii; Marshfield Clinic Health System, Marshfield, Wisconsin; and the University of Pennsylvania Health System, Philadelphia, Pennsylvania) that conducts research to better understand how to improve the cancer screening processes in community health care settings.^4,15^ The PROSPR Lung Consortium developed a retrospective cohort of patients aged 35 to 89 years who engaged with any of these 5 health care systems from January 1, 2010, through September 30, 2019. This study followed Strengthening the Reporting of Observational Studies in Epidemiology (STROBE) reporting guideline and was approved by the Kaiser Permanente Colorado Institutional Review Board, which waived the informed consent requirement because this observational study presented minimal risks to the participants whose data were analyzed.

### Data Sources and Cohort Identification

The PROSPR Lung Consortium common data model includes harmonized data on patient sex, race and ethnicity, smoking history, procedures, diagnoses, cancer registry, and Census tract-based measures of SES that were derived from each health care system’s administrative, electronic health record (EHR), and claims systems. To address variations of the changes associated with the 2021 USPSTF recommendations in the PROSPR Lung Consortium population compared with the CISNET estimates, we obtained self-reported measures of race and ethnicity from the individual patient's EHR. As shown in the Figure, we initially identified 2 overlapping cohorts of patients within the common data model who had complete smoking history and were engaged with the health care system for 12 or more continuous months as of September 30, 2019; we excluded those who had never smoked or who had unknown smoking history.

**Figure.**
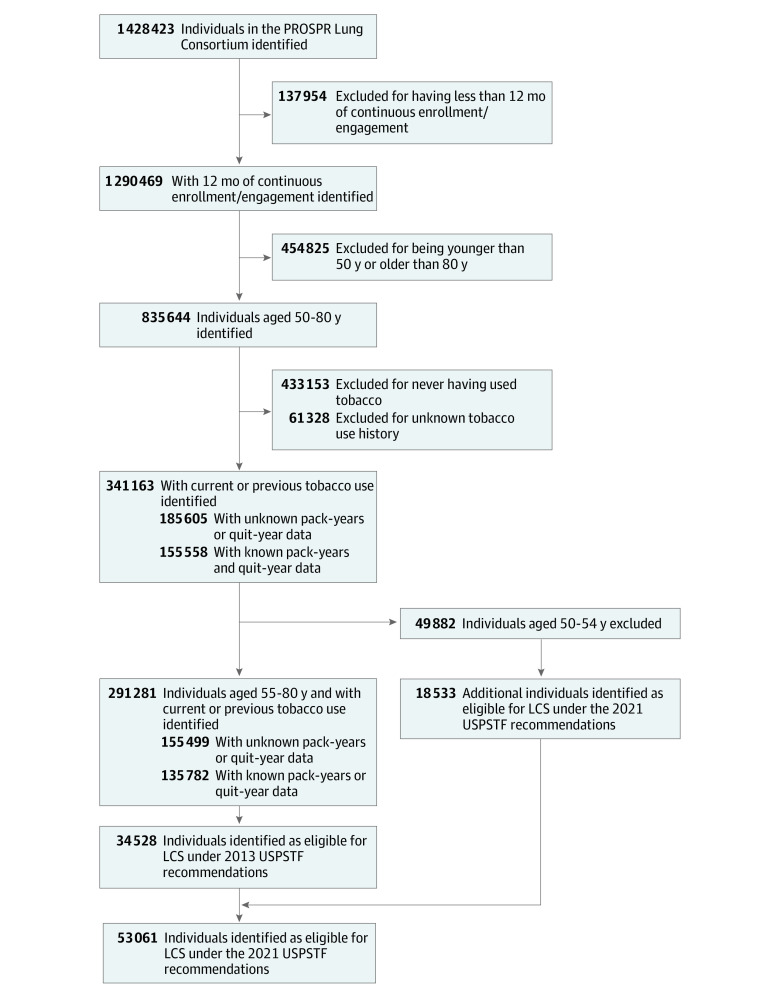
Profile of Individuals in the Population-based Research to Optimize the Screening Process (PROSPR) Lung Consortium Population and Eligibility for Lung Cancer Screening (LCS) Under the 2013 and 2021 US Preventive Services Task Force (USPSTF) Recommendations Cohorts

Specifically, the eligible cohort under the 2013 USPSTF recommendations included individuals aged 55 to 80 years who had at least a 30 pack-year smoking history and currently smoked or quit within the past 15 years, whereas the 2021 USPSTF recommendations’ eligible cohort included those aged 50 to 80 years who had at least a 20 pack-year smoking history and currently smoked or quit within the past 15 years. The 2021 eligible population also included individuals who were newly eligible under the expanded recommendations (eg, those aged 50-55 years and with a 20-29 pack-year smoking history). The 2021 newly eligible cohort represents the nonoverlapping comparator group compared with the 2013 USPSTF recommendations cohort.

Sensitivity analyses included individuals with incomplete smoking history (eg, only age-eligible current and previous smokers). To examine the potential increase in lung cancer identification by screening, we used site-level cancer registry data to identify patients aged 50 to 80 years with an incident lung cancer diagnosis between January 1, 2014, and September 30, 2019.

### Statistical Analysis

We described the distributions of age, sex, tobacco use, race and ethnicity, Charlson Comorbidity Index,^16^ chronic obstructive pulmonary disease diagnosis, SES (measured by the Yost Index^17,18^), and previous cancer for the 2013 and 2021 USPSTF recommendations’ eligible cohorts and for the nonoverlapping comparator group that would be newly eligible under the 2021 criteria. We described the relative changes in these variables in both the newly eligible population and the overall 2021 eligible cohort. Differences in the distribution of variables between the 2013 and newly eligible 2021 cohorts were evaluated with 2-sided χ^2^ tests. A 2-sided *P* < .05 was considered statistically significant. Analyses were performed with SAS statistical software, version 9.4 (SAS Institute Inc).

## Results

On September 30, 2019, we identified 1 428 423 individuals aged 35 to 89 years who received care within the 5 health care systems in the PROSPR Lung Consortium (Figure). Limiting the population to individuals who had 12 or more months of continuous enrollment or engagement, were between 50 and 80 years of age, and currently or previously smoked tobacco resulted in a population of 341 163 (eTable in the Supplement). Further limiting this population to those aged 55 to 80 years and applying the tobacco history exclusions yielded a final eligible cohort under the 2013 USPSTF recommendations of 34 528 individuals. The 2021 USPSTF recommendations added 18 533 individuals who were eligible for lung cancer screening for a total of 53 061 individuals, which was a relative increase of 53.7% compared with the 2013 USPSTF recommendations (Figure).

Table 1 describes the individual-level characteristics of the eligible cohorts for the 2013 and 2021 USPSTF recommendations as well as the newly eligible cohort under the 2021 USPSTF recommendations (nonoverlapping population). The 2021 newly eligible population included 5833 individuals (31.5%) aged 50 to 54 years, a larger proportion of women than men (52.0% [n = 9631] vs 48.0% [n = 8901]), and more racial or ethnic minority groups than the 2013 cohort. The relative increase for women was 13.8% higher than for men (61.2% vs 47.4%). The relative increase by race and ethnicity in the screening eligibility was 20.7% for non-Hispanic Black individuals compared with non-Hispanic White individuals (69.7% vs 49.0%). Similarly, the relative eligibility increase for Hispanic individuals was 18.4% (67.4% vs 49.0%) and for Asian, Hawaiian, or Pacific Islander individuals was 11.6% (60.6% vs 49.0%) compared with non-Hispanic White individuals.

**Table 1.** Characteristics of Eligible Cohorts Under the USPSTF 2013 and 2021 Lung Cancer Screening Recommendations

Characteristic	2013 USPSTF recommendations’ eligible cohort, No. (%)^a^	2021 USPSTF recommendations’ newly eligible cohort, No. (%)^b^	*P* value^c^	Relative increase, %	Overall 2021 USPSTF recommendations’ eligible cohort^b^
Total	34 528	18 533		53.7	53 061
Age at time of eligibility, y					
50-54	0	5833 (31.5)	<.001	NA	5833 (11.0)
55-59	6922 (20.0)	3052 (16.5)		44.1	9974 (18.8)
60-64	8905 (25.8)	3356 (18.1)		37.7	12 261 (23.1)
65-74	14 598 (42.3)	4869 (26.3)		33.4	19 467 (36.7)
75-80	4103 (11.9)	1423 (7.7)		34.7	5526 (10.4)
Sex					
Female	15 734 (45.6)	9631 (52.0)	<.001	61.2	25 365 (47.8)
Male	18 794 (54.4)	8901 (48.0)		47.4	27 695 (52.2)
Race and ethnicity					
Asian, Native Hawaiian, or Pacific Islander	1939 (5.6)	1174 (6.3)	<.001	60.6	3113 (5.9)
Hispanic	1279 (3.7)	862 (4.7)		67.4	2141 (4.0)
Multirace, other race, or unknown race^d^	1750 (5.1)	979 (5.3)		55.9	2729 (5.1)
Non-Hispanic Black	4929 (14.3)	3437 (18.5)		69.7	8366 (15.8)
Non-Hispanic White	24 631 (71.3)	12 081 (65.2)		49.0	36 712 (69.2)
Charlson Comorbidity Index score					
0	14 608 (42.3)	10 039 (54.2)	<.001	68.7	24 647 (46.5)
1	7290 (21.1)	3515 (19.0)		48.2	10 805 (20.4)
2	4708 (13.6)	2021 (10.9)		42.9	6729 (12.7)
≥3	7922 (22.9)	2958 (16.0)		37.3	10 880 (20.5)
COPD diagnosis	7720 (22.4)	2238 (12.1)	<.001	29.0	9958 (18.8)
Yost Index quintiles					
Q1	6228 (18.0)	3804 (20.5)	<.001	61.1	10 032 (18.9)
Q2	6273 (18.2)	3570 (19.3)		56.9	9843 (18.6)
Q3	7215 (20.9)	3717 (20.1)		51.5	10 932 (20.6)
Q4	7406 (21.4)	3708 (20.0)		50.1	11 114 (20.9)
Q5	6477 (18.8)	3214 (17.3)		49.6	9691 (18.3)
Missing data	929 (2.7)	520 (2.8)		56.0	1449 (2.7)
Center or site					
1	8244 (23.9)	4143 (22.4)	<.001	50.3	12 387 (23.3)
2	2511 (7.3)	1384 (7.5)		55.1	3895 (7.3)
3	14 202 (41.1)	7368 (39.8)		51.9	21 570 (40.7)
4	6837 (19.8)	3627 (19.6)		53.0	10 464 (19.7)
5	2734 (7.9)	2011 (10.9)		73.6	4745 (8.9)

Abbreviations: COPD, chronic obstructive pulmonary disease; NA, not applicable; USPSTF, US Preventive Services Task Force.

^a^ Participants aged 55 to 80 years with a history of 30 pack-years and current smoker or quit smoking within past 15 years.

^b^ Participants aged 50 to 80 years with a history of 20 pack-years and current smoker or quit smoking within past 15 years.

^c^ Pearson χ^2^ test of difference in characteristic distribution for people added under 2021 criteria vs 2013 criteria.

^d^ Multirace was a self-reportable option in the electronic health data. Other was an explicit race category available for reporting in one or more of the Healthcare Systems included in this study.

The 2021 newly eligible population had less comorbid illness, with the greatest relative increase of 68.7% in individuals with a Charlson Comorbidity Index score of 0. Similarly, the proportion of individuals with a diagnosis of chronic obstructive pulmonary disease in the newly eligible cohort was 12.1%, resulting in a decrease in the overall prevalence of chronic obstructive pulmonary disease from 22.4% (n = 7720) in the 2013 eligible cohort to 18.8% (n = 9958) in the 2021 cohort. In addition, the 2021 USPSTF recommendations were associated with a greater increase in newly eligible individuals in the lowest SES category. Specifically, 3804 newly eligible individuals (20.5%) were identified in Yost Index quintile 1 (lowest SES) compared with 3214 individuals (17.3%) within quintile 5 (highest SES), resulting in a relative increase of 61.1% for quintile 1 and 49.6% for quintile 5 (Table 1).

The sensitivity analysis that included all age-eligible individuals who currently or previously smoked also demonstrated significant and consistent changes in the distribution of the expanded eligibility for lung cancer screening by sex, race and ethnicity, and comorbidity status from those in the 2013 USPSTF recommendations cohort, but the differences were less pronounced (eTable in the Supplement). For example, the relative increase by race and ethnicity in the screening eligibility was 2.1% for non-Hispanic Black individuals compared with non-Hispanic White individuals (17.1% vs 16.9%).

Table 2 describes the characteristics of patients who were diagnosed with an incident lung cancer between January 1, 2014, and September 30, 2019, met the 2013 USPSTF recommendations, and theoretically would be newly eligible under the 2021 criteria, and the overall cohort who met the 2021 USPSTF criteria. Under the 2021 USPSTF recommendations for expanded eligibility, the identification of incident lung cancers increased by 30.0% (n = 379) compared with the 2013 USPSTF recommendations (n = 1265). The increase in lung cancer identification was most pronounced among women, racial and ethnic minority groups, and those with lower SES. For example, the largest relative increase (47.0%) by race and ethnicity was observed in non-Hispanic Black individuals, whereas the smallest increase (26.2%) occurred among non-Hispanic White individuals.

**Table 2.** Lung Cancer Cases Diagnosed in the PROSPR Lung Consortium Population Who Met the USPSTF 2013 and 2021 Lung Cancer Screening Recommendations

Characteristic	2013 USPSTF recommendations’ eligible cohort, No. (%)^a^	2021 USPSTF recommendations’ newly eligible cohort, No. (%)^b^	*P* value^c^	Relative increase, %	Overall 2021 USPSTF recommendations’ eligible cohort, No. (%)^b^
Total	1265	379		30.0	1644
Age group, y					
50-54	0	58 (15.3)	<.001	NA	58 (3.5)
55-59	126 (10.0)	38 (10.0)		30.2	164 (10.0)
60-64	253 (20.0)	54 (14.2)		21.3	307 (18.7)
65-74	636 (50.3)	162 (42.7)		25.5	798 (48.5)
75-80	250 (19.8)	67 (17.7)		26.8	317 (19.3)
Sex					
Female	632 (50.0)	229 (60.4)	<.001	36.2	861 (52.4)
Male	633 (50.0)	150 (39.6)		23.7	783 (47.6)
Race and ethnicity					
Asian, Native Hawaiian, or Pacific Islander	81 (6.4)	27 (7.1)	.003	33.3	108 (6.6)
Hispanic	40 (3.2)	11 (2.9)		27.5	51 (3.1)
Non-Hispanic Black	185 (14.6)	87 (23.0)		47.0	272 (16.5)
Non-Hispanic White	925 (73.1)	242 (63.9)		26.2	1167 (71.0)
Multirace, other race, or unknown race^d^	34 (2.7)	12 (3.2)		35.3	46 (2.8)
Center or site					
1	484 (38.3)	145 (38.3)	.93	30.0	629 (38.3)
2	369 (29.2)	109 (28.8)		29.5	478 (29.1)
3	117 (9.2)	35 (9.2)		29.9	152 (9.2)
4	44 (3.5)	10 (2.6)		NA	54 (3.3)
5	251 (19.8)	80 (21.1)		31.9	331 (20.1)

Abbreviations: NA, not applicable; PROSPR, Population-based Research to Optimize the Screening Process; USPSTF, US Preventive Services Task Force.

^a^ Participants aged 55 to 80 years with a history of 30 pack-years and current smoker or quit smoking within past 15 years.

^b^ Participants aged 50 to 80 years with a history of 20 pack-years and current smoker or quit smoking within past 15 years.

^c^ Pearson χ^2^ test of difference in characteristic distribution for people added under the 2021 criteria vs 2013 criteria.

^d^ Multirace was a self-reportable option in the electronic health data. Other is an explicit race category available for reporting in one or more of the Healthcare Systems included in this study.

## Discussion

To our knowledge, this study is the first in the community-based health care setting to examine both population and cancer identification changes associated with the 2021 USPSTF expansion of the lung cancer screening eligibility criteria. Consistent with the CISNET simulation and other studies,^8,9,11,19^ we estimated whether the increase in lung cancer screening eligibility under the 2021 USPSTF recommendations was larger for women than men; individuals in lower SES categories; and individuals who self-identified as having Asian, Native Hawaiian, or Pacific Islander, Hispanic, or non-Hispanic Black rather than non-Hispanic White race or ethnicity. We identified similar increases by sex, race and ethnicity, and SES in the proportion of patients with lung cancer who were eligible for screening under the 2021 USPSTF recommendations. Although estimates from the 2015 nationally representative survey data suggested that the 87% increase in eligibility was attributable to the 2021 recommendations,^11^ our estimates, which used EHR-derived demographic and clinical measures for patients in the PROSPR Lung Consortium cohort, suggested an overall eligibility increase of 53.7%. However, the methods and data sources used in the present evaluation of the 2021 USPSTF recommendations expansion differed substantially from those used by CISNET and other survey-based studies.

The findings of this study, in conjunction with those of CISNET, also indicated that health care systems should plan to increase lung cancer screening capacity by about 50% to 60% to accommodate the eligible cohort of the expanded 2021 recommendations. Considerations for adding lung cancer screening capacity should include screening coordinators to provide shared decision-making and manage future screening and evaluation, access to computed tomography scans and trained radiologists, and constraints related to access to thoracic surgeons for lung cancer resection surgery.^20^ Improvements in uptake and adherence to annual screening also play a role in increasing this capacity. Screening programs will need to closely monitor capacity strain and allocate resources appropriately to meet evolving demands as the 2021 USPSTF recommendations are adopted in clinical practice.

Findings of the present study suggest that expanding the USPSTF recommendations for lung cancer screening eligibility is an important step toward minimizing disparities in lung cancer screening, but health care systems will still need to invest substantial resources to tailor outreach strategies and reduce barriers to lung cancer screening uptake for those with lower SES and for racial and ethnic minority groups. Ultimately, the value of the expanded guidelines will be realized only if lung cancer screening rates are increased in high-risk and traditionally underserved populations, particularly among non-Hispanic Black individuals, who have some of the highest rates of lung cancer mortality in the US.

### Limitations

This study has several limitations. First, the PROSPR Lung Consortium cohort included a high proportion of current or former smokers whose EHR data did not capture pack-years and cessation date. This missing information reflects real-world health care system data capture on these components of lung cancer screening eligibility and highlights the need for clinician and system incentives to improve information capture to ascertain eligibility.^21^ This issue may be particularly relevant for underserved populations, those with lower health care use, and former smokers for whom the identification of smoking history is key to establishing eligibility.

Second, we used the Yost index, a well-validated, composite Census tract proxy for SES rather than self-reported measures of income, wealth, or other key social determinants of health to identify the potential economic, financial, or resource-based disparities associated with the 2021 USPSTF recommendations.

Third, we did not address the potential benefits and harms of the expanded eligibility for lung cancer screening within and across the PROSPR Lung Consortium. Specifically, we did not examine the potential changes per-screen rate of false-positives that were associated with the 2021 USPSTF recommendations. Currently, no US data are available on false-positive rates in the expanded patient population (aged 50-54 years or with less tobacco history), making it difficult to estimate the false-positives with any certainty. The CISNET modeling estimate for per-screen rate of false-positives for the 2021 USPSTF recommendations was 11.86%,^11^ which was consistent with the 2013 USPSTF recommendations’ eligibility estimates of 12.8% from the NLST^22^ and 10.4% from an analyses of a diverse, urban, underserved, community-based lung cancer screening program.^23^ Despite these limitations, the dynamic and frequently updated data infrastructure of the PROSPR Lung Consortium, which is based on individuals who receive health care services in community settings, provided insight into how the 2021 USPSTF recommendations may help reduce the disparities among individuals who are eligible for lung cancer screening in the United States.

## Conclusions

This cohort study showed that the 2021 USPSTF recommendations were associated with increased lung cancer screening eligibility of women, racial and ethnic minority individuals, and those with lower SES who were eligible for such a screening. This updated eligibility criteria may help reduce barriers to screening access for individuals at highest risk for lung cancer.

## Notes

Supplement. **eTable.** Characteristics of PROSPR-Lung Population, USPSTF-2013 Versus USPSTF-2021 Age Recommendations

## References

[1] Aberle DR, Adams AM, Berg CD, ; National Lung Screening Trial Research Team. Reduced lung-cancer mortality with low-dose computed tomographic screening. N Engl J Med. 2011;365(5):395-409. doi:10.1056/NEJMoa1102873 21714641 PMC4356534

[2] Carroll NM, Burnett-Hartman AN, Joyce CA, . Real-world clinical implementation of lung cancer screening-evaluating processes to improve screening guidelines-concordance. J Gen Intern Med. 2020;35(4):1143-1152. doi:10.1007/s11606-019-05539-w 31974902 PMC7174472

[3] Gould MK, Sakoda LC, Ritzwoller DP, . Monitoring lung cancer screening use and outcomes at four cancer research network sites. Ann Am Thorac Soc. 2017;14(12):1827-1835. doi:10.1513/AnnalsATS.201703-237OC 28683215 PMC5711261

[4] Kim R, Rendle KA, Neslund-Dudas CM, . Community-based lung cancer screening adherence to Lung-RADS recommendations. Am J Clin Oncol. 2021;39:10540. doi:10.1200/JCO.2021.39.15_suppl.10540

[5] Neslund-Dudas CM, Tang A, Alleman E, . Completion of lung cancer screening after a baseline order for LDCT at five diverse health systems. Am J Clin Oncol. 2021;39:10506. doi:10.1200/JCO.2021.39.15_suppl.10506

[6] Zahnd WE, Eberth JM. Lung cancer screening utilization: a behavioral risk factor surveillance system analysis. Am J Prev Med. 2019;57(2):250-255. doi:10.1016/j.amepre.2019.03.015 31248742

[7] Haddad DN, Sandler KL, Henderson LM, Rivera MP, Aldrich MC. Disparities in lung cancer screening: a review. Ann Am Thorac Soc. 2020;17(4):399-405. doi:10.1513/AnnalsATS.201907-556CME 32017612 PMC7175982

[8] Pasquinelli MM, Tammemägi MC, Kovitz KL, . Risk prediction model versus United States Preventive Services Task Force lung cancer screening eligibility criteria: reducing race disparities. J Thorac Oncol. 2020;15(11):1738-1747. doi:10.1016/j.jtho.2020.08.006 32822843

[9] Pinsky PF, Lau YK, Doubeni CA. Potential disparities by sex and race or ethnicity in lung cancer screening eligibility rates. Chest. 2021;160(1):341-350. doi:10.1016/j.chest.2021.01.070 33545164 PMC8411441

[10] Fiscella K, Winters P, Farah S, Sanders M, Mohile SG. Do lung cancer eligibility criteria align with risk among Blacks and Hispanics? PLoS One. 2015;10(11):e0143789. doi:10.1371/journal.pone.0143789 26618478 PMC4664289

[11] Meza R, Jeon J, Toumazis I, . Evaluation of the benefits and harms of lung cancer screening with low-dose computed tomography: modeling study for the US Preventive Services Task Force. JAMA. 2021;325(10):988-997. doi:10.1001/jama.2021.1077 33687469 PMC9208912

[12] Jonas DE, Reuland DS, Reddy SM, . Screening for lung cancer with low-dose computed tomography: updated evidence report and systematic review for the US Preventive Services Task Force. JAMA. 2021;325(10):971-987. doi:10.1001/jama.2021.0377 33687468

[13] Doria-Rose VP, White MC, Klabunde CN, . Use of lung cancer screening tests in the United States: results from the 2010 National Health Interview Survey. Cancer Epidemiol Biomarkers Prev. 2012;21(7):1049-1059. doi:10.1158/1055-9965.EPI-12-0343 22573798 PMC3392469

[14] Miller EA, Pinsky PF. Healthcare access, utilization, and preventive health behaviors by eligibility for lung cancer screening. J Cancer Educ. 2021;36(2):330-337. doi:10.1007/s13187-019-01634-y 31656025

[15] Rendle KA, Burnett-Hartman AN, Neslund-Dudas C, . Evaluating lung cancer screening across diverse healthcare systems: a process model from the Lung PROSPR Consortium. Cancer Prev Res (Phila). 2020;13(2):129-136. doi:10.1158/1940-6207.CAPR-19-0378 31871221 PMC7010351

[16] Johnstone MS, McMillan DC, Horgan PG, Mansouri D. The relationship between co-morbidity, screen-detection and outcome in patients undergoing resection for colorectal cancer. World J Surg. 2021;45(7):2251-2260. doi:10.1007/s00268-021-06079-3 33774690 PMC8154830

[17] Yu M, Tatalovich Z, Gibson JT, Cronin KA. Using a composite index of socioeconomic status to investigate health disparities while protecting the confidentiality of cancer registry data. Cancer Causes Control. 2014;25(1):81-92. doi:10.1007/s10552-013-0310-1 24178398

[18] Boscoe FP, Liu B, Lee F. A comparison of two neighborhood-level socioeconomic indexes in the United States. Spat Spatiotemporal Epidemiol. 2021;37:100412. doi:10.1016/j.sste.2021.100412 33980407

[19] Aldrich MC, Mercaldo SF, Sandler KL, Blot WJ, Grogan EL, Blume JD. Evaluation of USPSTF lung cancer screening guidelines among African American adult smokers. JAMA Oncol. 2019;5(9):1318-1324. doi:10.1001/jamaoncol.2019.1402 31246249 PMC6604090

[20] Blom EF, Ten Haaf K, Arenberg DA, de Koning HJ. Treatment capacity required for full-scale implementation of lung cancer screening in the United States. Cancer. 2019;125(12):2039-2048. doi:10.1002/cncr.32026 30811590 PMC6541509

[21] Modin HE, Fathi JT, Gilbert CR, . Pack-year cigarette smoking history for determination of lung cancer screening eligibility: comparison of the electronic medical record versus a shared decision-making conversation. Ann Am Thorac Soc. 2017;14(8):1320-1325. doi:10.1513/AnnalsATS.201612-984OC 28406708

[22] Pinsky PF, Gierada DS, Black W, . Performance of Lung-RADS in the National Lung Screening Trial: a retrospective assessment. Ann Intern Med. 2015;162(7):485-491. doi:10.7326/M14-2086 25664444 PMC4705835

[23] Kaminetzky M, Milch HS, Shmukler A, . Effectiveness of Lung-RADS in reducing false-positive results in a diverse, underserved, urban lung cancer screening cohort. J Am Coll Radiol. 2019;16(4, pt A):419-426. doi:10.1016/j.jacr.2018.07.011 30146484

