# Supplementary material for: Evaluation of Population-Level Changes Associated With the 2021 US Preventive Services Task Force Lung Cancer Screening Recommendations in Community-Based Health Care Systems
Source: JAMA Netw Open. 2021 Oct 12;4(10):e2128176. doi: 10.1001/jamanetworkopen.2021.28176 (PMC8511972; doi:10.1001/jamanetworkopen.2021.28176)
Supplement: Supplement. — eTable. Characteristics of PROSPR-Lung Population, USPSTF-2013 Versus USPSTF-2021 Age Recommendations [file jamanetwopen-e2128176-s001.pdf]

Supplementary Online Content

Ritzwoller DP, Meza R, Carroll NM, et al. Evaluation of population-level changes associated with the 2021 US Preventive Services Task Force lung cancer screening recommendations in community-based health care systems. *JAMA Netw Open*. 2021;4(10):e2128176. doi: 10.1001/jamanetworkopen.2021.28176

eTable. Characteristics of PROSPR-Lung Population, USPSTF-2013 Versus USPSTF-2021 Age Recommendations

This supplementary material has been provided by the authors to give readers additional information about their work.

© 2021 Ritzwoller DP et al. *JAMA Network Open.*

eTable. Characteristics of PROSPR-Lung Population, USPSTF-2013 Versus USPSTF-2021 Age Recommendations

Population Characteristics	USPSTF-2013	Newly Eligible Individuals added by USPSTF-2021 Age Criteria			USPSTF-2021
	Total Population Age 55-80 AND CURRENT or PREVIOUS TOBACCO USE N (%)	N (%)	p-value*	Relative Increase (%)	Total Population Age 50-80 AND CURRENT or PREVIOUS TOBACCO USE N (%)
**Total N**	291,281	49,882		17.1	341,163
**Age at time of Eligibility**					
50 - 54	0 (0.0)	49,882	<.001	N/A	49,882 (14.6)
55 - 59	62,143 (21.3)	0		0	62,143 (18.2)
60 - 64	68,693 (23.6)	0		0	68,693 (20.1)
65 - 74	115,824 (39.4)	0		0	115,824 (33.9)
75 - 80	44,621 (15.3)	0		0	44,621 (13.1)
**Sex**					
Female	146,629 (50.3)	25,395 (50.9)	0.02	17.1	172,024 (50.4)
Male	144,648 (49.7)	24,485 (49.1)		16.9	169,133 (49.6)
**Race/Ethnicity**					
Non-Hispanic Black	48,947 (16.8)	8,588 (17.2)	<.001	17.5	57,535 (16.9)
Hispanic	12,919 (4.4)	3,001 (6.0)		23.2	15,920 (4.7)
Asian/ Native Hawaiian/ Pacific Islander	19,533 (6.7)	4,262 (8.5)		21.8	23,785 (12.9)
Multi-race/ Other/Unknown	16,909 (5.8)	3,227 (6.5)		19.1	20,136 (5.9)
Non-Hispanic White	192,973 (66.2)	30,804		16.0	223,777 (65.6)
**Charlson Comorbidity Score**					
0	162,130 (55.7)	36,884 (74.0)	<.001	22.8	199,018 (58.3)
1	50,984 (17.5)	7,523 (15.1)		14.8	58,507 (17.1)
2	32,721 (11.2)	3,073 (6.2)		9.4	35,794 (10.5)

© 2021 Ritzwoller DP et al. *JAMA Network Open.*

3+	45,446 (15.6)	2,398 (4.8)		5.3	47,844 (14.0)
**COPD Diagnosis**	22,829 (7.8)	1,536 (3.1)	<.001	5.5	24,093 (7.1)
**Yost Index quintiles**					
Q1	47,137 (16.2)	8,443 (16.9)	<.001	17.9	55,580 (16.3)
Q2	47,081 (16.2)	8,668 (17.4)		18.4	55,749 (16.3)
Q3	55,077 (18.9)	9,707 (19.5)		17.6	64,784 (19.0)
Q4	61,695 (21.2)	10,413 (20.9)		16.9	72,108 (21.1)
Q5	71,220 (24.5)	11,063 (22.2)		15.5	82,283 (24.1)
Missing	9,071 (3.1)	1,588 (3.2)		17.5	10,659 (3.1)
**Center**					
Site 1	61,497 (21.1)	17,491 (35.1)	<.001	17.1	70,424 (20.6)
Site 2	25,874 (8.9)	8,927 (17.9)		14.5	30,699 (9.0)
Site 3	102,462 (35.2)	4,825 (9.7)		18.6	119,953 (35.2)
Site 4	67,542 (23.2)	6,634 (13.3)		19.6	79,547 (23.3)
Site 5	33,906 (11.6)	12,005 (24.1)		17.8	40,540 (11.9)

* Pearson Chi-square test of difference in characteristic distribution for people added by 2021 criteria vs. 2013 criteria

© 2021 Ritzwoller DP et al. *JAMA Network Open.*
